# Supplementary material for: Evolution of optimal growth temperature in Asgard archaea inferred from the temperature dependence of GDP binding to EF-1A
Source: Nat Commun. 2024 Jan 15;15:515. doi: 10.1038/s41467-024-44806-1 (PMC10789797; doi:10.1038/s41467-024-44806-1)
Supplement: Supplementary file 1 — Supplementary Information [file 41467_2024_44806_MOESM1_ESM.pdf]

Fig. S1 Phylogenetic analysis of archaeal and eukaryotic EF-1A, and bacterial EF-Tu.

The maximum likelihood phylogenetic trees were inferred based on best-fitting empirical (LG+F+I+G4) and mixture (Q.pfam+C40+F+R6) models, respectively. Both the trees are rooted on the bacterial EF-Tu, and bootstrap values are shown for some nodes.

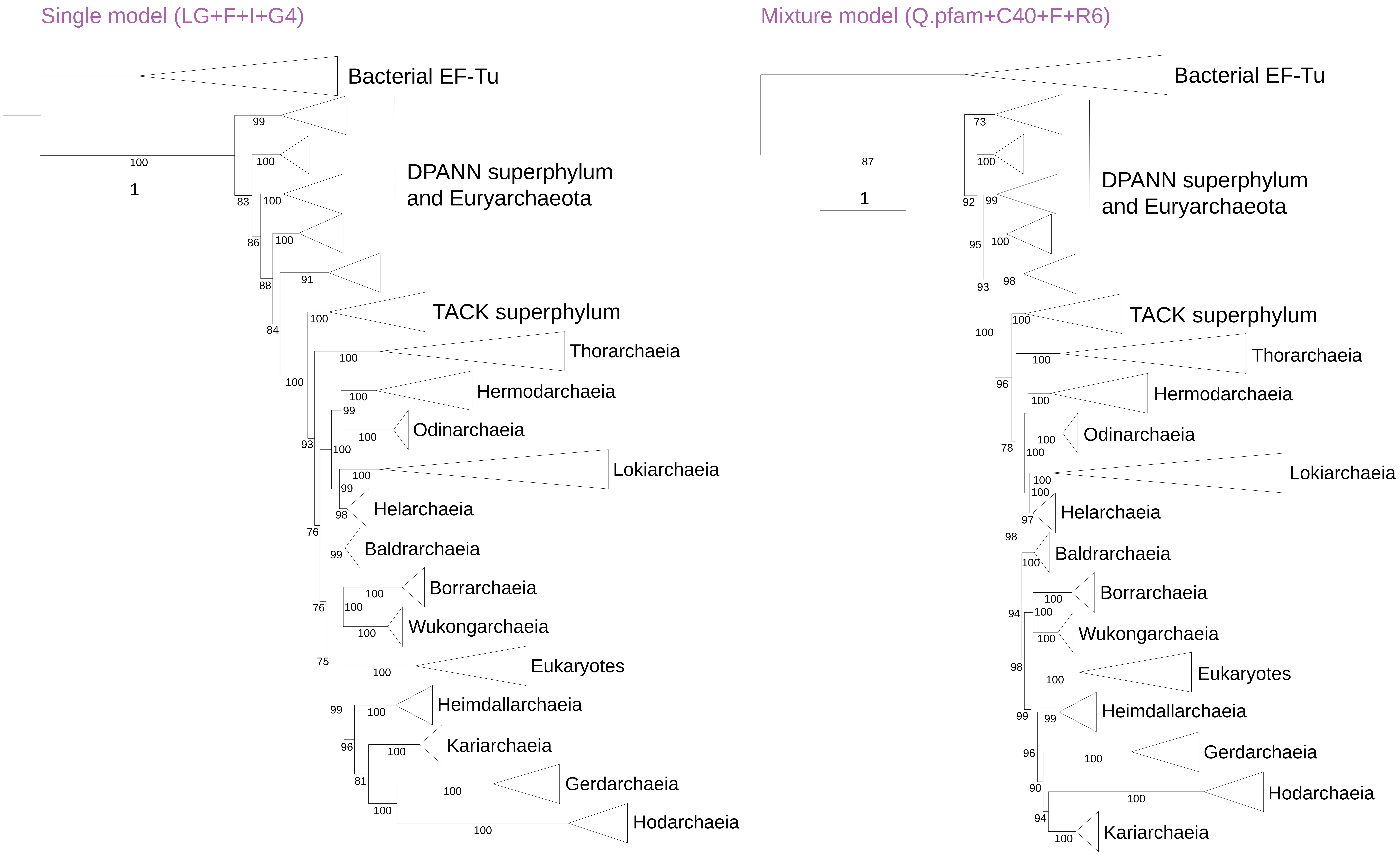

Fig. S2 Maximum likelihood phylogeny of archaeal and eukaryotic EF-1A, and bacterial EF-Tu.
The tree was reconstructed by maximum likelihood analysis using 195 amino acid sequences based on best-fitting model LG+F+I+G4 model, with option "-bb 1000", using IQ-tree. The tree is rooted on bacterial EF-Tu, and the bootstrap values were shown on nodes.

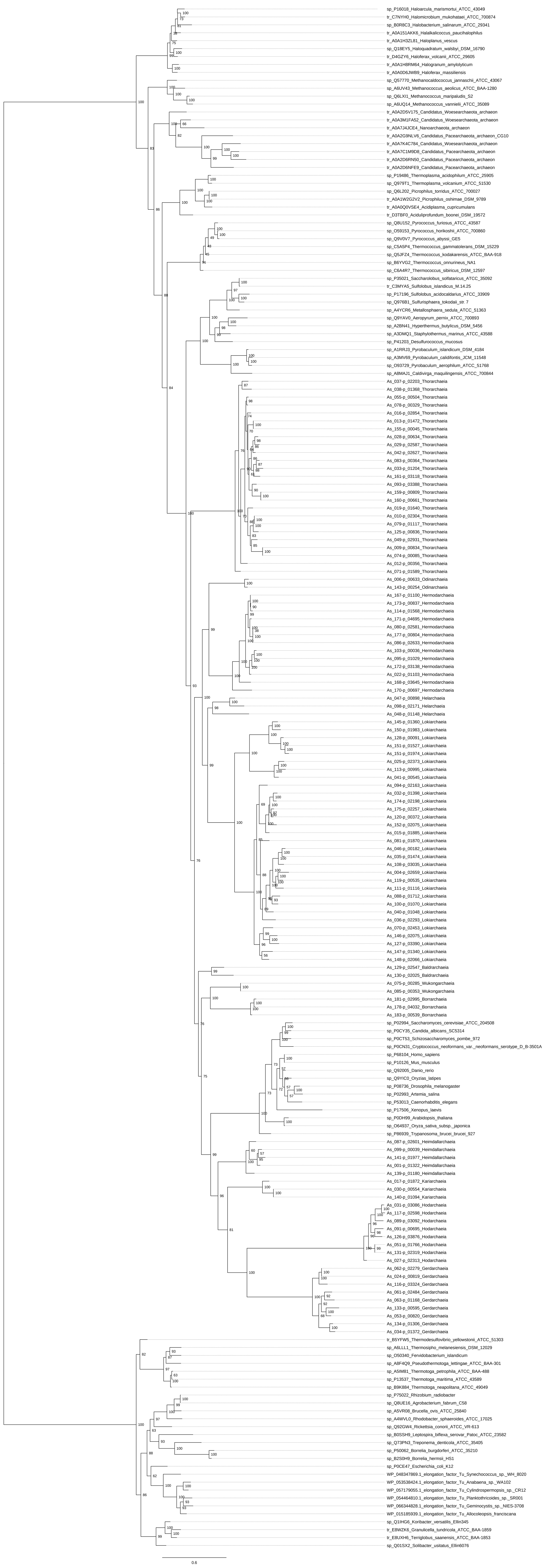

Fig. S3 Thermal stability range of the EF-1A/Tu and ancestral EF-1A.
The highest fluorescence intensity (a. u., arbitrary units) reflects that substantial protein unfolding occurred. Source data are provided as a Source Data file.

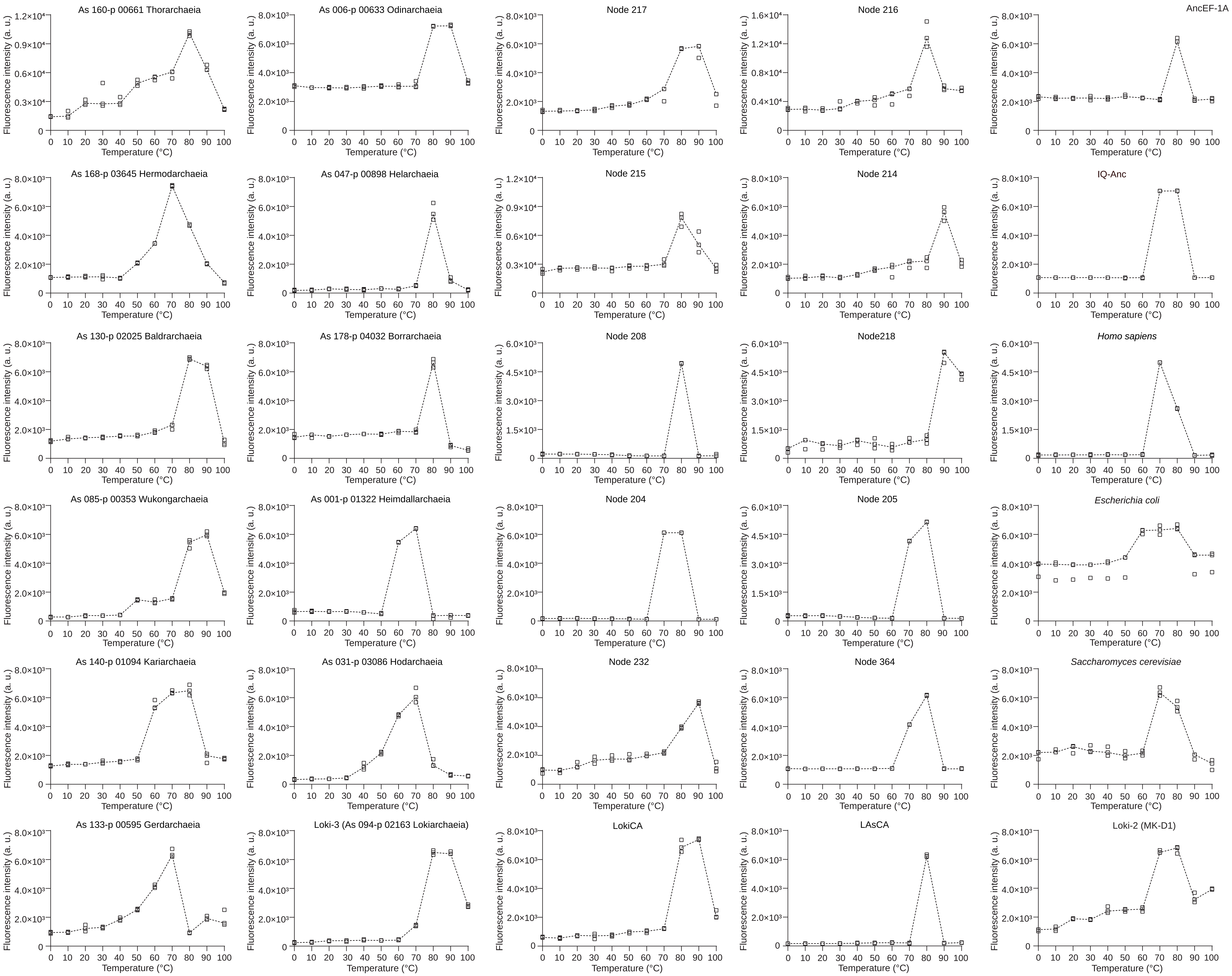

Fig. S4 Statistical analysis of the optimal GDP-binding temperature of the EF-1A/Tu and ancestral EF-1A. The testing EF-1A/Tu and ancestral EF-1A proteins were labeled with color according to their optimal temperature for GDP binding. Source data are provided as a Source Data file.

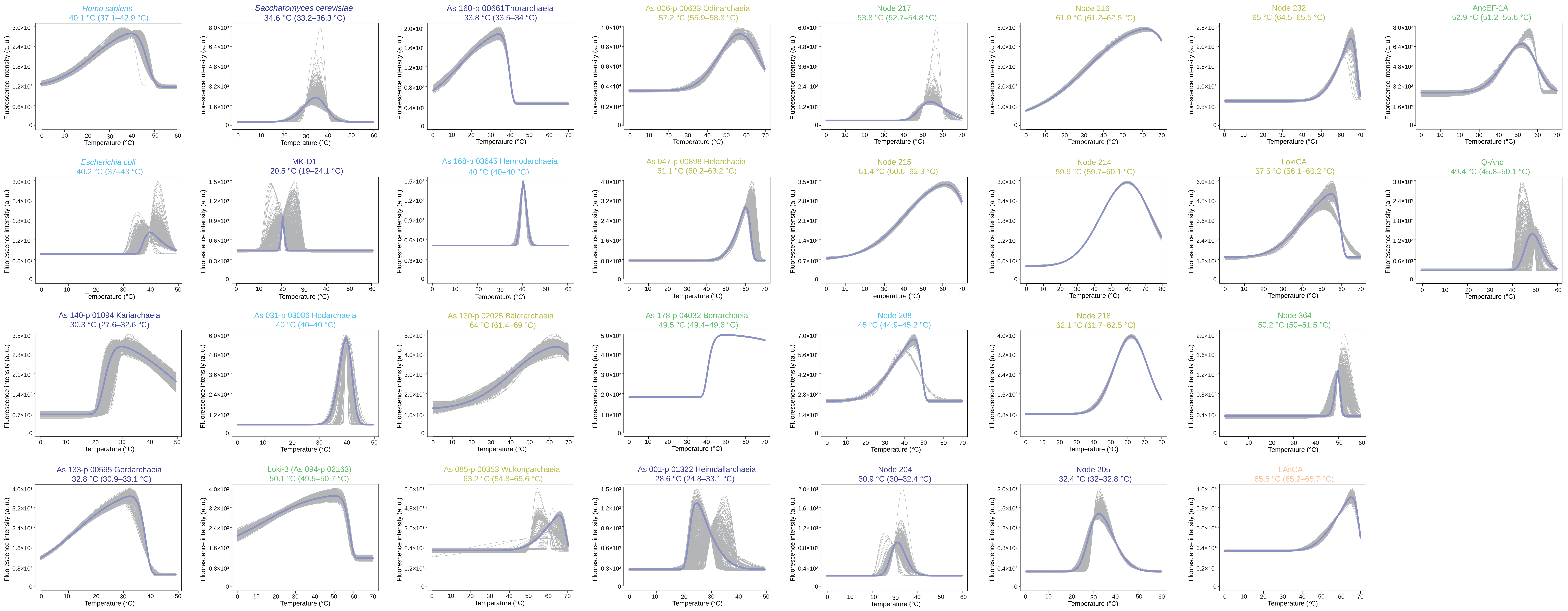

Fig. S5 Inferring evolutionary trajectory of optimal growth temperature of Asgard archaea based on the optimal GDP-binding temperature of EF-1A proteins. (a) Evolutionary of the Asgard and eukaryotic EF-1A proteins. The tree is a schematic representation according to maximum likelihood phylogeny of Asgard and eukaryotic EF-1A in Fig. S2. The testing EF-1A proteins were labeled with color according to their optimal temperature for GDP binding. Divergent evolution of Lokiarchaeia subgroup Loki-2 (blue) and Loki-3 (green) was highlighted. MK-D1 refers to Candidatus 'Prometheoarchaeum syntrophicum' strain MK-D1.
(b) Evaluation of optimal GDP-binding temperature of the EF-1A/Tu and ancestral EF-1A proteins used in this study. Source data are provided as a Source Data file.

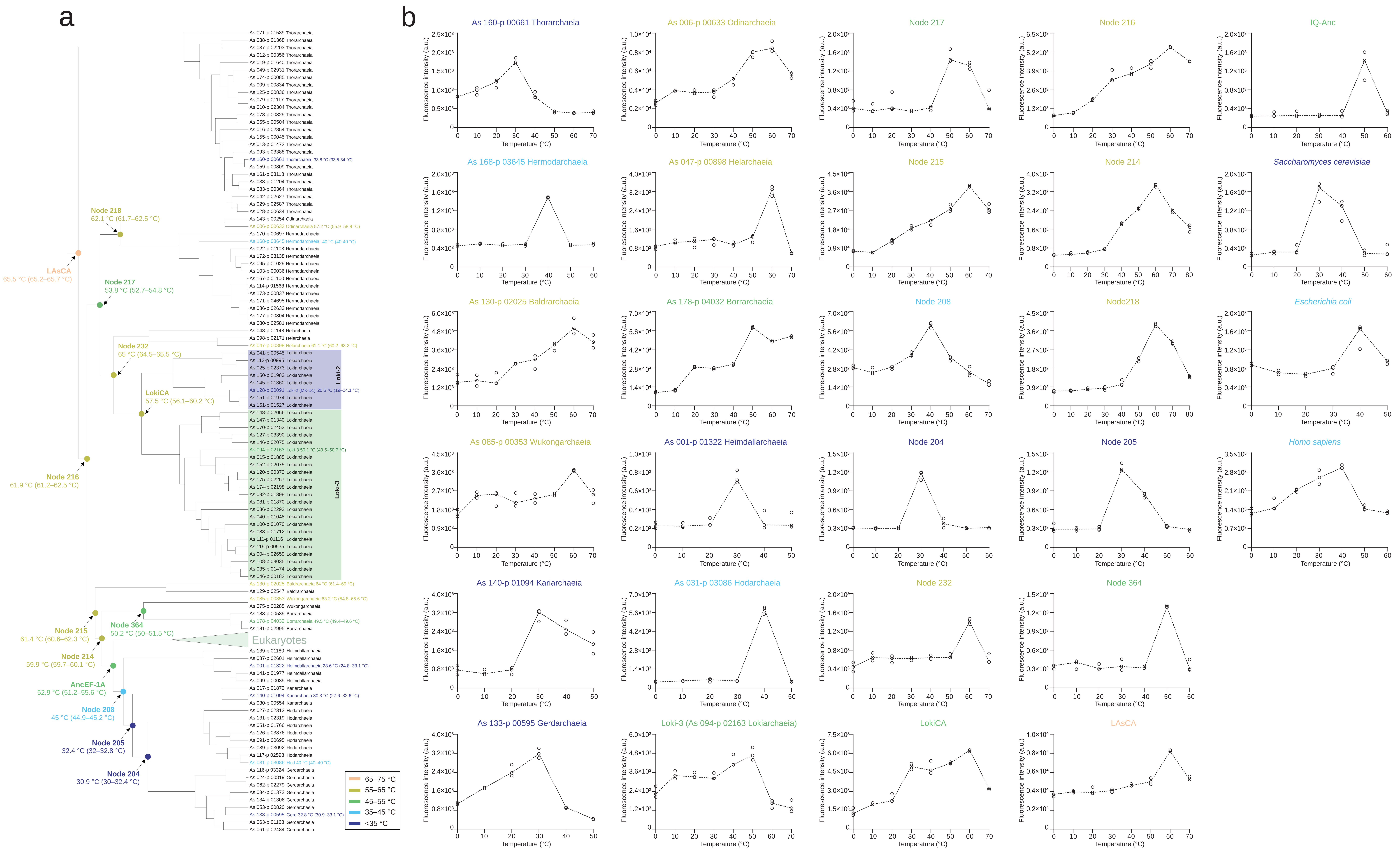

Fig. S6 Alignment of amino acid sequences of Asgard EF-1A. To trim the amino acid sequences of Asgard EF-1A, the N- and C-terminal residues (labeled in red) were removed. The average values of amino acid sequence similarity of the trimmed EF-1A within Asgardarchaeota phylum and Lokiarchaeia subgroups, and between the Lokiarchaeia subgroups were labeled in blue and red, respectively. The canonical EF-1A domain (PRK12317) was labeled.

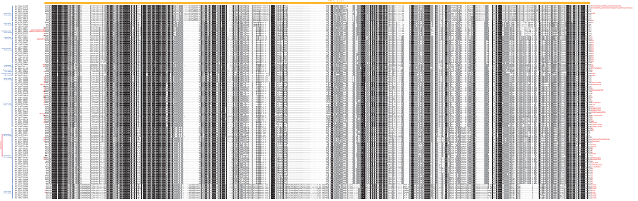

Fig. S7 Correlation analysis between GDP-binding temperature and genomic-based inferred Asgard OGTs.
(a) Correlation analysis between GDP-binding temperature and our inferred Asgard OGTs. (b) Correlation analysis between GDP-binding temperature and the Asgard OGTs predicted by reference 4. Both panels a and b were subject to t-test, and the p value was less than 0.0001, which met the conditions for correlation analysis. Source data are provided as a Source Data file

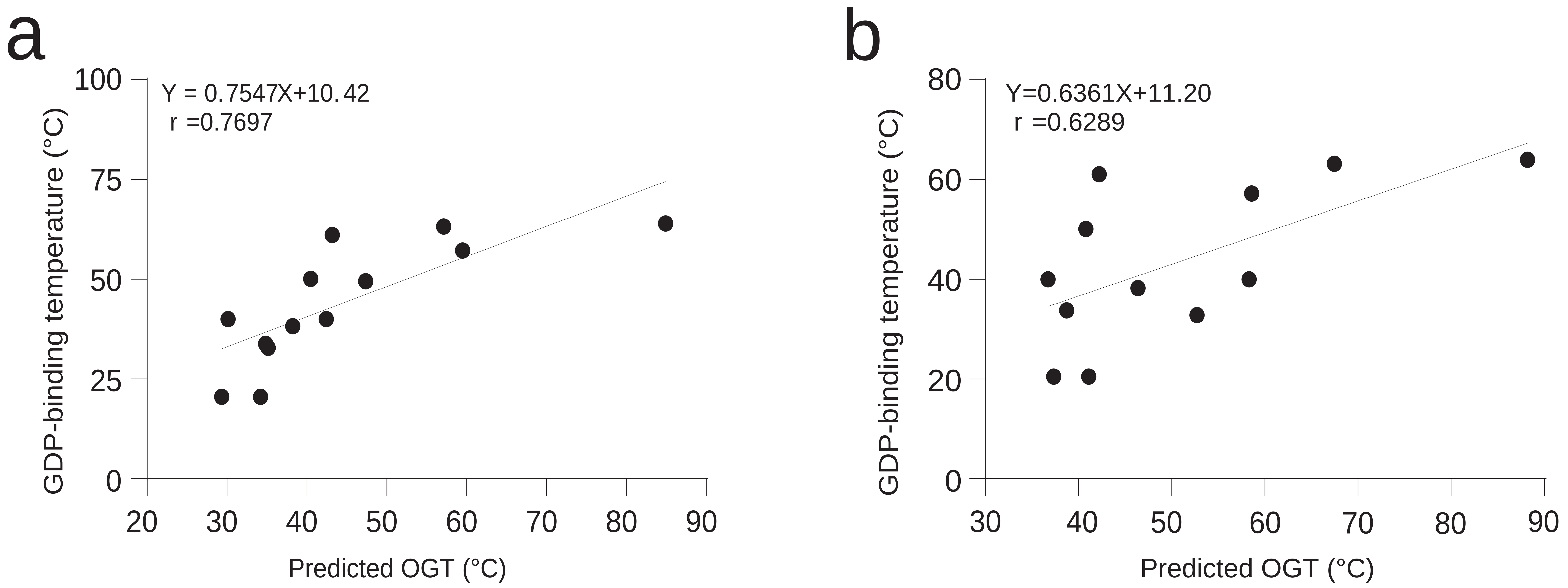

Fig. S8 Amino acid sequences of ancestral EF-1A proteins used here. The Posterior probabilities (PP) for amino acid states of the ancestral EF-1A are labeled.

```
Loki CA  : MAKDKPHLNLVIIGHIDHGKSTMMGHLLIKTGAVSDREARELEKLAQELDRESWKYAYVFDRLKEERQRGITIDLAFRKFETKNYYFTIIDAPGHADFVKNMITGASQADAAILVVSGKKGEFEAGIS-------------ANGQTREHAFLAKTLGVKQLVVAINKMD--TWNYSEDRYNEVKEEVSDLLKNV-- : 179
LAsCA    : --MSKPHLNLVIIGHVDHGKSTMMGRLLYETGAIDERTIRKFEEEAEKLGKESFKFAWILDRLKEERERGLTIDLAFRKFETKKYYFTIIDAPGHRDFIKNMITGASQADAAILVVSAKKGEFEAGIG-------------PGGQTREHAFLARTLGVNQLIVAVNKMDDSTVNYSQERYNEIKEEVSDFLKSI-- : 179
Node205  : MSKEKPHLNLVIIGHVDHGKSTMTGHLLYLCGAIDERTIEKYAKESEKMGKGSWKYAWVLDKLKEERERGLTIDIAFFKFETKKYYFTIIDAPGHRDFVKNMITGTSQADAAILVVSAEKGEFEAGIS-------------AEGQTREHALLAKTLGVNQLIVAVNKMDDPTVDWSQERYNEIKEEVSGRLKQF-- : 181
Node208  : MSKEKPHLNLVIIGHVDHGKSTMTGHLLYLCGAIDERTIEKFEKEAEKMGKGSFKYAWVLDKLKEERERGLTIDLAFFKFETKKYYFTIIDAPGHRDFVKNMITGTSQADAAILVVSAEKGEFEAGIS-------------AEGQTREHALLAKTLGVNQLIVAVNKMDDPTVDWSQERYNEIKEEVSGRLKQF-- : 181
Node214  : MSKEKPHLNLVIIGHVDHGKSTLMGHLLYLTGAIDERTIRKFEEEAEKLGKGSFKYAWVLDKLKEERERGLTIDLAFWKFETKKYYFTIIDAPGHRDFVKNMITGTSQADAAILVVSAKKGEFEAGIG-------------PGGQTREHAFLAKTLGVNQLIVAVNKMDDPTVNWSQERYNEIKEEVSGLLKQI-- : 181
Node215  : MSKEKPHLNLVIIGHVDHGKSTLMGHLLYLTGAIDERTIRKFEEEAEKLGKGSFKYAWVLDKLKEERERGLTIDLAFWKFETKKYYFTIIDAPGHRDFVKNMITGASQADAAILVVSAKKGEFEAGIG-------------AGGQTREHAFLAKTLGVNQLIVAVNKMDDPTVNWSQERYNEIKEEVSGLLKQI-- : 181
Node216  : MSKEKPHLNLVIIGHVDHGKSTLMGHLLYLTGAIDERTIRKLEEEAEKLGKGSFKYAWVLDRLKEERERGLTIDLAFRKFETKKYYFTIIDAPGHRDFVKNMITGASQADAAILVVSAKKGEFEAGIG-------------AGGQTREHAFLARTLGVNQLIVAVNKMDDPTVNWSQERYNEIKEEVSDLLKQI-- : 181
Node364  : MSKEKPHLNLVIIGHVDHGKSTLMGHLLYLTGAIDERTIRKFEEEAEKLGKGSFKYAWVLDKLKEERERGLTIDLAFWKFETKKYYFTIIDAPGHRDFVKNMITGTSQADAAILVVSAKKGEFEAGIG-------------KGGQTVEHAVLAATLGVNQFIVAVNKMDDPTVNWSQDRYNEIKSNVDKLIKER-- : 181
Node217  : MSEKKPHLNLVIIGHVDHGKSTLMGHLLYLTGAISDREMRKLEKMAQKLDKGSFKYAFVLDRLKEERERGLTIDLAFRKFETKKYYFTIIDAPGHRDFVKNMITGASQADAAILVVSAKKGEFEAGIS-------------AGGQTREHAFLARTLGVNQLIVAVNKMDDPTVNWSQERYNEIKEEVSDLLKQV-- : 181
Node218  : MSEKKPHLNLVIIGHVDHGKSTLMGHLLYKTGAISDREMRKLEKLAQKLDKGSFKYAFVMDRLKEERERGLTIDLAFRKFETQKYYFTIIDAPGHRDFVKNMITGASQADAAILVVSARKGEFEAGIS-------------AGGQTREHAFLARTLGVNQLIVAVNKMDDSTVNWSQERYNEIKEEVSGLLEGK-- : 181
Node232  : MSEKKPHLNLVIIGHVDHGKSTMMGHLLILTGAISDREMRELEKMAQKLDKGSFKYAFVFDRLKEERERGLTIDLAFRKFETKKYYFTIIDAPGHRDFVKNMITGASQADAAILVVSAKKGEFEAGIS-------------AGGQTREHAFLARTLGVNQLIVAVNKMDDPTVNWSQERYNEIKEEVSDLLKQV-- : 181
Node204  : MSKEKPHLNLVIIGHVDHGKSTMTGHLLFLCGAIDERTIEKYAKESEKMGKGSWKYAWVLDKLKEERERGLTIDISFFKFETKKYVFTIIDAPGHRDFVKNMITGASQADAAILVVSAEKGEFEAGISAERGTPKGDWRIPGGQTREHALLAKTLGVNQLIVAVNKMD--TVDWSQERYNEIKEEVSALLKQFVE : 193

Loki CA  : --GYPVE----KIPFVPVSALTGDNLTEKSTTLPWYDGPTLIEALDQFELPEKPTDKPLRLPIQDVYKIKGAGVVPVGRVETGVLKVGDKVIIMPTGY-----FGEVRSIEMHHEEMPQAEPGDNIGFNIRGITMKDVHRGDVVGHA-DNPPTVVTPNGNFTGQIIVIW----------H-PTAIAQGYTPVVHA : 351
LAsCA    : --GYKVD----KIPFIPVSAWTGDNLTEKSPNMPWYDGPTLLEALDTFEVPPKPIDKPLRIPIQDVYSITGVGTVPVGRVETGVLKVGDKVIFMPPGV-----TGEVKSIEMHHEQMPQAEPGDNIGFNVRGIEKKDIKRGDVAGHV-DNPPTVAE---EFTGQIIVIY----------H-PTAIAVGYTPVIHA : 348
Node205  : --GYDLD----KVPFVPVSGMTGDNLTDKSVTLPWYDGPTLLEALDTFEVPPKPTDKPLRLPIQDVYSITGIGTVPVGRVETGVLKPGDKVIFAPSGG-----KAEVKSIEMHHEQLPQAEPGDNIGFNVKGIAKKDIRRGDVVGHPGDNPPKVAK---TFTATVIVIK----------G-PTAIAAGYTPVIHC : 351
Node208  : --GYDLD----KVPFVPVSGMTGDNLTEKSVTVPWYDGPTLLEALDTFEVPPKPIDKPLRLPIQDVYSITGIGTVPVGRVETGVLKPGDKVIFAPSGY-----KAEVKSIEMHHEQLPQAEPGDNIGFNVKGISKKDIRRGDVVGHPGDNPPTVAK---EFTATVIVIK----------G-PTAIAAGYTPVIHC : 351
Node214  : --GYDLD----KVPFVPVSAWTGDNLTEKSVTVPWYDGPTLLEALDTFEVPPKPIDKPLRIPIQDVYSITGVGTVPVGRVETGVLKVGDKVIFMPSGL-----TGEVKSIEMHHEQLQQAEPGDNIGFNVKGISKKDIRRGDVAGHPGDNPPTVAK---EFTGQIIVIY----------H-PTAIAAGYTPVIHA : 351
Node215  : --GYDLD----KVPFVPVSAWTGDNLTEKSVTVPWYDGPTLLEALDTFEVPPKPIDKPLRIPIQDVYSITGVGTVPVGRVETGVLKVGDKVIFMPPGL-----TGEVKSIEMHHEQLQQAEPGDNIGFNVKGISKKDIRRGDVAGHP-DNPPTVAK---EFTGQIIVIY----------H-PTAIAAGYTPVIHA : 350
Node216  : --GYDVD----KVPFIPVSAWTGDNLTEKSVTVPWYDGPTLLEALDTFEVPPKPIDKPLRIPIQDVYSITGVGTVPVGRVETGVLKVGDKVIFMPPGL-----TGEVKSIEMHHEQLQQAEPGDNIGFNVRGISKKDIKRGDVAGHV-DNPPTVAE---EFTGQIIVIY----------H-PTAIAAGYTPVIHA : 350
Node364  : --ELDKK----KVPYVPVSAWTGDNLTEKSVTVPWYDGPTLLEALDTFEVPPKPIDKPLRIPIQDVYSITGVGTVPVGRVETGVLKVGDKVIFMPSGG-----KGEVKSIEMHHEQLQKAEPGDNIGFNVKGISKKDIRRGDVAGHP-DNPPTVAK---EFTGQIFVIY----------HPPTAIAAGYTPVIHA : 351
Node217  : --GFPVD----KVPFIPVSAWTGDNLTEKSTTMPWYDGPTLLEALDTFELPPKPIDKPLRIPIQDVYSITGVGTVPVGRVETGVLKVGDKVIFMPPGV-----TGEVKSIEMHHEQLQQAEPGDNIGFNVRGISKKDIKRGDVAGHV-DNPPTVVE---EFTGQIIVIY----------H-PTAIAAGYTPVIHA : 350
Node218  : --GYDVD----KIPFIPVSAWTGDNLTEPSVTVPWYKGPTLLEALDTFELPPKPVDKPLRIPIQDVYTITGVGTVPVGRVETGVLKVGDKVIFMPPGV-----TGEVKSIEMHHERLEQAEPGDNIGFNVRGIGKGDIKRGDVAGHV-DNPPTVVE---EFTGQIIVIH----------H-PTAIAAGYTPVIHA : 350
Node232  : --GFPVD----KVPFIPVSAWTGDNLTEKSVTVPWYDGPTLLEALDTFELPPKPVDKPLRLPIQDVYSITGVGTVPVGRVETGVLKVGDKVIFMPPGL-----TGEVRSIEMHHEQLQQAEPGDNIGFNIRGISKKDIKRGDVAGHV-DNPPTVVE---EFTGQIIVIY----------H-PTAIAPGYTPVIHA : 350
Node204  : GSGYRLDGKGLGVKFVPTSGMTGDNLTDKSDKMPWYDGPSLLEALDTFEVPEKPTDKPLRLPIQDVYSITGIGTVPVGRVESGVLKPGDKVIFAPSKYGIKPLTAEVKSIEMHHEQLPQAEPGDNIGFNVKGIAKKDILRGDVVGHPGDNPPKVAD---TFTATVIVIKGLGKPGKKANHPPTGIAAGYTPVIHC : 385

Loki CA  : HTAQVACKFVELSKKLDPKTGQVIEDNPQFLKKNDAAIVKLQPIKKLCLEKYKDFPELGRFAVRDMGRTVAVGVVKDIETG : 432  mean posterior probability (PP) =0.90
LAsCA    : HTAQVACRFTELISKLDPRTGQVVEENPDFIKQGDAAIVKLKPIRPMVIEKYSDFPQLGRFAVRDMGRTVAVGVVIDVKPK : 429  mean PP =0.92
Node205  : GTAQVACRFAEIKEKIDRRTGQVKEANPDFLKNGDRATVVMRPVRPMVVEKFSDFPQLGRFAVRDMGQTVAVGIVKDVQPK : 432  mean PP =0.88
Node208  : GTAQVACRFAELKSKIDRRTGQVKEANPDFLKNGDAATVVMVPTRPMVVEKYSDFPQLGRFAVRDMGQTVAVGIVKDVTPK : 432  mean PP =0.91
Node214  : HTAQVACRFAELKSKIDRRTGQVVEENPDFLKSGDAAIVKLVPTRPMVIEKYSDFPQLGRFAVRDMGRTVAVGIVKDVTPK : 432  mean PP =0.93
Node215  : HTAQVACRFAELISKIDRRTGQVVEENPDFLKQGDAAIVKFVPTRPMVIEKYSDFPQLGRFAVRDMGRTVAVGIVKDVTPK : 431  mean PP =0.94
Node216  : HTAQVACRFEELISKIDPRTGQVVEENPDFLKQGDAAIVKFKPIRPMVIEKYSDFPQLGRFAVRDMGRTVAVGVVKDVTPK : 431  mean PP =0.94
Node364  : HTAQVACTFAELQSKIDRRTGQVVEENPDYLKSGDGAIVKLVPTRPMVIEKYSDFPQLGRFAVRDMGRTVAVGIVKDVTPK : 432  mean PP =0.92
Node217  : HTAQVACKFEELISKIDPRTGQVIQEKPDFLKQGDAAIVKFKPIRPLVIEKYSDFPQLGRFAVRDMGRTVAVGVVKEVTPK : 431  mean PP =0.92
Node218  : HTAQVACKFTELISKIDPRTGQVIQEKPDFIKQGDAAIVKFKPIRPLVIEKYSDFPQLGRFAVRDMGRTVAVGVVKEVTPK : 431  mean PP =0.91
Node232  : HTAQVACKFEELIQKIDPRTGQVIQEKPDFLKQGDAAIVKFKPIRPLVIEKYSDFPQLGRFAVRDMGRTVAVGVVKEVKPK : 431  mean PP =0.92
Node204  : GTAQVACRFA IKEKIDRRTGQVKEANPDFLKQGDRATVVMRPVRPMVVEKFSDFPQLGRFAVRDMGQTVAVGIVKDVQPK : 466  mean PP =0.81
```
