# Supplementary material for: Evolution of optimal growth temperature in Asgard archaea inferred from the temperature dependence of GDP binding to EF-1A
Source: Nat Commun. 2024 Jan 15;15:515. doi: 10.1038/s41467-024-44806-1 (PMC10789797; doi:10.1038/s41467-024-44806-1)
Supplement: Supplementary file 3 — Description of Additional Supplementary Files [file 41467_2024_44806_MOESM3_ESM.pdf]

Description of Additional Supplementary Files:

Supplementary Data 1: Statistical analysis of the optimal GDP-binding temperature of Asgard EF-1A proteins. Topt, calculated optimal GDPbinding temperature; q05, 5th percentile of the peak temperature distribution; q95, 95th percentile of the peak temperature distribution.

Supplementary Data 2: Prediction of optimal growth temperature of Asgard archaea based on the proteomic features. The sample collection environments for metagenomics are listed.

Supplementary Data 3: Amino acid sequences of ancestral Asgard EF-1A used in this study.
