# Supplementary material for: Evolution of optimal growth temperature in Asgard archaea inferred from the temperature dependence of GDP binding to EF-1A
Source: Nat Commun. 2024 Jan 15;15:515. doi: 10.1038/s41467-024-44806-1 (PMC10789797; doi:10.1038/s41467-024-44806-1)
Supplement: Supplementary file 7 — Reporting Summary [file 41467_2024_44806_MOESM7_ESM.pdf]

nature portfolio

Corresponding author(s): Meng Li

Last updated by author(s): Dec 16, 2023

Reporting Summary

Nature Portfolio wishes to improve the reproducibility of the work that we publish. This form provides structure for consistency and transparency in reporting. For further information on Nature Portfolio policies, see our Editorial Policies and the Editorial Policy Checklist.

Statistics

For all statistical analyses, confirm that the following items are present in the figure legend, table legend, main text, or Methods section.

n/a	Confirmed	
☐	☒	The exact sample size (*n*) for each experimental group/condition, given as a discrete number and unit of measurement
☒	☐	A statement on whether measurements were taken from distinct samples or whether the same sample was measured repeatedly
☐	☒	The statistical test(s) used AND whether they are one- or two-sided<br>*Only common tests should be described solely by name; describe more complex techniques in the Methods section.*
☒	☐	A description of all covariates tested
☒	☐	A description of any assumptions or corrections, such as tests of normality and adjustment for multiple comparisons
☐	☒	A full description of the statistical parameters including central tendency (e.g. means) or other basic estimates (e.g. regression coefficient) AND variation (e.g. standard deviation) or associated estimates of uncertainty (e.g. confidence intervals)
☐	☒	For null hypothesis testing, the test statistic (e.g. *F*, *t*, *r*) with confidence intervals, effect sizes, degrees of freedom and *P* value noted<br>*Give P values as exact values whenever suitable.*
☒	☐	For Bayesian analysis, information on the choice of priors and Markov chain Monte Carlo settings
☒	☐	For hierarchical and complex designs, identification of the appropriate level for tests and full reporting of outcomes
☐	☒	Estimates of effect sizes (e.g. Cohen's *d*, Pearson's *r*), indicating how they were calculated

Our web collection on statistics for biologists contains articles on many of the points above.

Software and code

Policy information about availability of computer code

Data collection	The data were collected using UniProt database (https://www.uniprot.org/), NCBI database (https://www.ncbi.nlm.nih.gov/), and our local Asgard archaea database (PMID: 33911286).
Data analysis	The MUSCLE (v3.8.1551) and IQ-tree (v1.6.5) were used for phylogenetic analysis of EF proteins. The IQ-tree (v1.6.5), FastML webserver (http://fastml.tau.ac.il/), and PAML (v4.9) were used for inferring ancestral protein sequences. The ColabFold server (https://colab.research.google.com/github/sokrypton/ColabFold/blob/main/AlphaFold2.ipynb) was used for predicting protein structure. The machine learning model Tome (v1.0) was used for predicting OGT based on genomes. The Gradprism (9.0.0) was used for calculating Pearson correlation coefficient r. The script to run the statistical analysis of the optimal GDP-binding temperature is available at Zenodo (https://zenodo.org/badge/latestdoi/703800685).The machine learning model Tome (v1.0) was used to predict OGT values of the Asgard archaea based on the proteomic features.

For manuscripts utilizing custom algorithms or software that are central to the research but not yet described in published literature, software must be made available to editors and reviewers. We strongly encourage code deposition in a community repository (e.g. GitHub). See the Nature Portfolio guidelines for submitting code & software for further information.

Data

Policy information about availability of data

All manuscripts must include a data availability statement. This statement should provide the following information, where applicable:

- Accession codes, unique identifiers, or web links for publicly available datasets
- A description of any restrictions on data availability
- For clinical datasets or third party data, please ensure that the statement adheres to our policy

The bioinformatic and biochemical data generated in this study have been deposited in Supplementary Information/Source Data file. Source data are provided with this paper. The script to run the statistical analysis of the optimal GDP-binding temperature is available at https://zenodo.org/badge/latestdoi/703800685.

Research involving human participants, their data, or biological material

Policy information about studies with human participants or human data. See also policy information about sex, gender (identity/presentation), and sexual orientation and race, ethnicity and racism.

Reporting on sex and gender	n/a
Reporting on race, ethnicity, or other socially relevant groupings	n/a
Population characteristics	n/a
Recruitment	n/a
Ethics oversight	n/a

Note that full information on the approval of the study protocol must also be provided in the manuscript.

Field-specific reporting

Please select the one below that is the best fit for your research. If you are not sure, read the appropriate sections before making your selection.

☐ Life sciences ☐ Behavioural & social sciences ☒ Ecological, evolutionary & environmental sciences

For a reference copy of the document with all sections, see nature.com/documents/nr-reporting-summary-flat.pdf

Ecological, evolutionary & environmental sciences study design

All studies must disclose on these points even when the disclosure is negative.

Study description	This work used ancestral sequence reconstruction and experimentally determine the optimal GDP-binding temperature of a translation elongation factor EF-1A from ancient and extant Asgard archaea, to explore OGT evolution of Asgard archaea and infer optimal growth temperatures of the last Asgard ancestor of eukaryotes.
Research sample	The research sample is 195 translation elongation factors including 106 EF-1A from our local Asgard database (PMID: 33911286) and the other archaeal, bacterial EF and eukaryotic protein from UniProt database (https://www.uniprot.org/) and NCBI database (https://www.ncbi.nlm.nih.gov/). These EF proteins were chosen because they are reviewed EF proteins that harbor a typical PRK12317 domain. These sample represent the EF of archaeal, bacterial, and eukaryotic EF proteins when explore the EF evolution. In particular, the other archaeal EF proteins were chosen covering representative archaeal lineages, which is sufficient for exploring EF evolution in archaea.
Sampling strategy	A total of 195 EF proteins were sampled in this work. We firstly collected the 106 EF proteins from our local Asgard database (PMID: 33911286). We then chosen the other reviewed archaeal, bacterial, and eukaryotic EF proteins from UniProt database (https://www.uniprot.org/) and NCBI database (https://www.ncbi.nlm.nih.gov/). The Asgard EF proteins are all we can collected based on our database, and they cover the major Asgard lineages. The other archaeal EF proteins cover the all known major archaeal phyla and superphyla. Thus, these archaeal EF proteins are sufficient for exploring EF evolution in archaea domain. Since this work focuses on Asgard EF evolution, the bacterial and eukaryotic EF proteins are sufficient for using as outgroup.
Data collection	Zhongyi Lu collected the EF protein sequences from UniProt database (https://www.uniprot.org/), NCBI database (https://www.ncbi.nlm.nih.gov/), and our local Asgard archaea database (PMID: 33911286) using computer. Zhongyi Lu collected the bioinformatic data (including phylogenetic analysis, protein structure construction, sequence alignment, and ancestral protein predication, OGT predication based on genomes) using computer. Runyue Xia recorded the protein purification using Bio-Rad Gel Doc XR, NanoDrop Spectrophotometers, and ÄKATA Pure Protein Purification System. Runyue Xia recorded the GDP-binding temperature data of EF proteins using Infinite 200 PRO and computer. Runyue Xia analyzed correlation between OGT and GDP-binding temperature of EF using computer.
Timing and spatial scale	The research sample is protein sequences that were deposited in our local database and online database (e.g. NCBI and Uniprot). This

	work focused on EF evolution just based on the protein sequences and biochemically characterized them. Neither timing nor spatial scale factor was required here.
Data exclusions	No data were excluded from the analyses.
Reproducibility	Triplicate measurements for the biochemical analyses presented.
Randomization	There was no data that required randomization.
Blinding	In this study, we collected the Asgard EF and other archaeal and bacterial proteins from our local and online database (PMID: 33911286). These EF proteins were all manually reviewed to ensure they belong to a same protein family, which is essential for phylogenetic analysis and inferring ancestral protein sequence. There was no data that required blinding.

Did the study involve field work? ☐ Yes ☒ No

Reporting for specific materials, systems and methods

We require information from authors about some types of materials, experimental systems and methods used in many studies. Here, indicate whether each material, system or method listed is relevant to your study. If you are not sure if a list item applies to your research, read the appropriate section before selecting a response.

Materials & experimental systems

n/a	Involved in the study	
☒	☐	Antibodies
☒	☐	Eukaryotic cell lines
☒	☐	Palaeontology and archaeology
☒	☐	Animals and other organisms
☒	☐	Clinical data
☒	☐	Dual use research of concern
☒	☐	Plants

Methods

n/a	Involved in the study	
☒	☐	ChIP-seq
☒	☐	Flow cytometry
☒	☐	MRI-based neuroimaging

Plants

Seed stocks	n/a
Novel plant genotypes	n/a
Authentication	n/a
